# Early detection of infectious bovine keratoconjunctivitis with artificial intelligence

**DOI:** 10.1186/s13567-023-01255-w

**Published:** 2023-12-15

**Authors:** Shekhar Gupta, Larry A. Kuehn, Michael L. Clawson

**Affiliations:** 1 MyAnIML, Overland Park, KS 66223 USA; 2 United States Department of Agriculture (USDA), Agricultural Research Service (ARS), U. S. Meat Animal Research Center, Clay Center, NE 68933 USA

**Keywords:** Artificial intelligence, neural networks, deep learning, biosecurity, cattle disease, animal welfare, infectious bovine keratoconjunctivitis, pinkeye, cattle, muzzles

## Abstract

Artificial intelligence (AI) was developed to distinguish cattle by their muzzle patterns and identify early cases of disease, including infectious bovine keratoconjunctivitis (IBK). It was tested on 870 cattle in four locations, with 170 developing IBK. The AI identified 169 of the 170 cases prior to their identification by veterinarians, and another 17 cases that remained free of IBK signs (sensitivity = 99.4%, specificity = 97.6%). These results indicate the AI can detect emerging IBK cases by muzzle images very early in the disease process and be used as an intervention tool in the prevention of IBK outbreaks.

## Introduction, methods, and results

Infectious bovine keratoconjunctivitis (IBK), also known as pinkeye, is the most important ocular disease of cattle in the United States and throughout much of the world [1, 2]. The disease is both a cattle welfare and economic problem [1, 3]. IBK is painful, with clinical signs that include lacrimation, blepharospasm, photophobia, epiphora, and serous or mucopurulent conjunctivitis [3, 4]. Corneas can become scarred and severe IBK cases can lead to permanent blindness [2, 3]. Cattle with IBK can have reduced weaning weights, incurring a substantial economic loss [1].

IBK is a disease complex that often involves bacterial species of *Moraxella*, including *Moraxella bovis*, which is a causative agent of IBK, and a specific genotype of *Moraxella bovoculi* (genotype 1) which associates with the disease but has not been shown to directly cause it [2, 5, 6]. Additional bacteria have been associated with IBK, or IBK-like disease in what appear to be more minor roles, including *Mycoplasma bovis*, *Mycoplasma bovoculi*, *Ureplasma* species, *Listeria monocytogenes*, and *Chlamydia* species, although the exact roles and strength of IBK associations for many of them is unclear [7]. Also, some viruses can cause ocular lesions in cattle including herpesvirus BoHV-1 and a γ-herpesvirus [7].

In the US, approved antibiotic treatments for IBK are oxytetracycline and the macrolide tulathromycin [8]. The treatments can be effective, however, they come at an economic cost, and their application in cattle raises concerns of a spike of antimicrobial resistance that could potentially spill over from cattle to humans or components of the environment [9, 10]. Given that, a primary goal for IBK is prevention [11]. There currently are no highly efficacious vaccines that protect cattle from IBK in the field, thus, there is a need for novel solutions to preventing IBK outbreaks in cattle [11, 12].

Like fingerprints of humans, muzzle dermatoglyphic patterns of cattle can identify the animals at the individual level [13, 14]. Features can be extracted from digital camera images with algorithms such as scale-invariant feature transform (SIFT) or maximally stable extremal regions (MSER), that can be used with machine learning, deep learning or convolutional neural networks for individual identification [15, 16]. The possibilities of muzzle dermatoglyphics with artificial intelligence applications in the early detection of diseases are only now beginning to be explored. In the case of IBK, early muzzle changes that proceed visible signs of disease have not been reported in the literature, but seemed possible prior to this study as the bovine cornea epithelium is densely but only superficially innervated [3], indicating that very early stages of IBK are likely painful to the animal, which in turn could affect the muzzle appearance. Early detection of animals about to break with IBK would be critically important for the development of interventions that prevent IBK outbreaks. Therefore, the goals of this study were to create a database of muzzle images of cattle with and without signs of IBK, to create a pipeline for image feature extraction, to train machine learning to differentiate between muzzle images of cattle with and without signs of IBK, and to test the ability of the artificial intelligence system to detect cattle with early, breaking cases of IBK before veterinarians could detect signs of the disease.

### Creation of a pipeline to identify sick cattle by images of their muzzles

**Figure 1:**
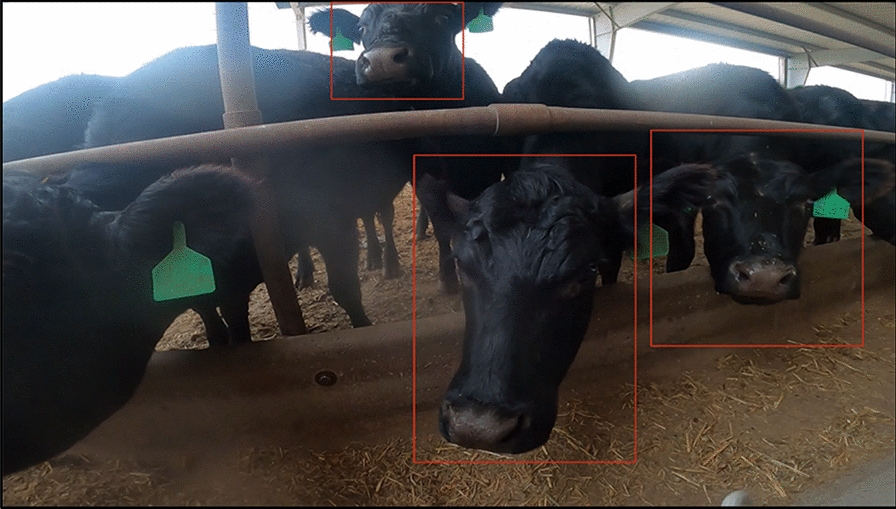
**Pictures of cattle taken with GoPro camera mounted on feeder truck**. The AI grabs individual images for analyses.

**Figure 2:**
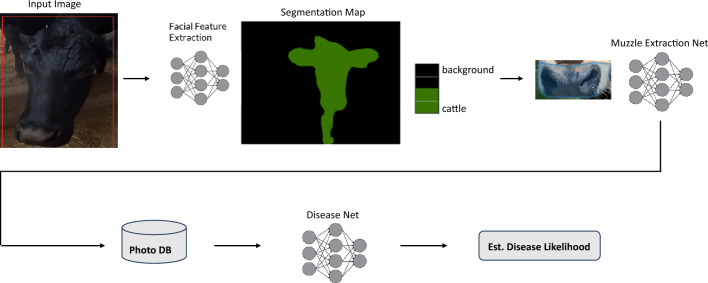
**Pipeline overview of the technology.** Every pixel of the image is classified as either pertaining to the individual cattle or not. Machine learning extracts facial features so the computer would know what is background and what is cattle. The system ends up knowing exactly which pixels belong to a particular animal. **A** bounding box is drawn around the pertinent part of the image and cropped out. This both improves model accuracy and reduces storage and processing costs.

The AI pipeline was made of two different models. The first model was designed to extract critical information about cattle muzzles using pictures. The second was designed to predict whether or not cattle were sick by comparing their pictures to those in MyAnIML’s large database of healthy and sick muzzles (Figures 1 and 2). For this study, we developed preliminary AI algorithms using Regional Convolutional Neural Networks (RCNNs and Pytorch). We developed our own RCNNs for object detection as well as image classification tasks across datasets. We used Sagemaker from AWS to do analyses on the cloud. We also compared Yolo3 with RCNN, Tensorflow, and Pytorch.

To identify and crop the muzzle from the pictures, the AI measured an animal’s face by measuring the distance from the left-to-right ear and forehead-to-lower chin (Figure 2). The AI would then create a “virtual box” of the individual’s face so that it could be analyzed (Figure 2). Once the system obtained facial feature extractions, it created a muzzle extraction net (Figure 2).

Since there were no cattle facial and muzzle libraries publicly available prior to this study, we developed one with the help of our partners. We captured 3000 healthy beef cattle pictures to train the AI. We also captured 1500 sick beef cattle pictures. Most of the cattle were crossbreeds. Out of 1500 sick cattle pictures, we had 800 IBK cases and 600 bovine respiratory disease (BRD) cases, and the rest were of different diseases such as foot rot, uterus infection, ect. The pictures were used to show the system the difference between healthy and sick muzzles.

### IBK study

An IBK study was conducted in the summers of 2021 (June–Aug) and 2022 (July and Aug). The cattle were Angus 40%, Hereford 35%, and Charolais 25%, with 55% mature animals, 25% calves, and 20% heifers. Muzzle pictures were taken in three different cattle backgrounder operations in the Kansas City area: Paola, Harrisonville, Garnett, and Kansas City, KS. Paola and Kansas City, KS were from one backgrounder with two different locations. The first set of pictures was taken in 2021 with either cell phones or digital cameras, and was of animals contained within a squeeze chute. The second set was taken in 2022 using a GoPro camera on a Feeder Truck (Figure 1). In 2021, 150 cattle were photographed in Paola and 220 were photographed in Kansas City (see Table 1 for cattle numbers and IBK results). In 2022, 200 cattle were photographed in Harrisonville and 300 cattle were photographed in Garnett. All 870 muzzle pictures were analyzed by our AI for the early detection of IBK. Additionally, all 870 animals in the study were examined for IBK by veterinarians the same day their pictures were taken, and 1–2 days after they were taken. Disease diagnoses were made by the veterinarians and none of the animals were diagnosed with IBK on the day their pictures were taken. The veterinarians were notified to check on cattle flagged by our AI system as having IBK in the interest of animal welfare. Of the 870 animals, 170 were diagnosed with IBK based on early signs including lacrimation with epiphora. The veterinarians also checked for signs of mechanical injury to the eyes which would have excluded an IBK diagnosis. Prior to their diagnosis, 169 of the 170 cases were identified as developing IBK by the AI (sensitivity = 99.4%). The AI identified another 17 animals that would develop IBK but remained negative during the study (specificity = 97.6%).

**Table 1:** **IBK study information**.

Paola cattle used in study	150
Kansas City cattle used in study	220
Harrisonville cattle used in study	200
Garnett cattle used in study	300
Total number of cattle in study	870
Total predicted healthy by AI that did not develop IBK	683
Total predicted healthy by AI that did develop IBK	1
Total predicted IBK cases by AI that did develop IBK	169
Total predicted IBK cases by AI that did not develop into IBK	17

Numbers of cattle from different locations used in the study and comparisons of IBK predictions by AI versus diagnosed cases.

## Discussion

Surveillance and early detection of infectious disease is a key component to disease management and outbreak control [17]. This is particularly true for diseases in which efficacious vaccines are lacking, like IBK. IBK can emerge quickly in cattle herds and is considered highly contagious, although the exact mechanisms by which *M. bovis*, *M. bovoculi*, and other IBK-associated bacteria spread from animal to animal are unclear [3]. Thus, the AI system developed in this study could be used as an intervention tool to prevent IBK outbreaks, given that it accurately predicted the emergence of IBK cases very early in the disease progression. Environmental practices used to control the spread or severity of IBK could be implemented earlier through AI, such as enhanced fly control protocols, greater shading for protection from ultraviolet light, and disinfection between the processing or handling of cases [18, 19]. Infected animals could also be separated from their uninfected herd mates when possible. Additionally, the early identification of cases could lead to judicious administration of antibiotics before a large outbreak had the chance to emerge.

There are limitations to the system in its current stage of development. The AI was trained exclusively on beef cattle, thus additional training with dairy cattle muzzles would be necessary prior to its use for IBK detection in dairy cattle. Additional data on beef cattle could be used to train the system further, and potentially detect any specific breed effects regarding muzzle profiles with or without IBK. Visual inspection of the muzzles of cattle predicted to develop IBK in this study did not reveal a clear phenotype that correlated exclusively with the disease, thus the biological differences between the muzzles of healthy beef cattle versus those of emerging IBK cases are not fully understood. Notably, *M. bovis* and *M. bovoculi* strain types that associate with IBK can all carry the repeats-in-toxin hemolysin [6, 20], which is considered one of their main virulence factors [21]. It is possible these different organisms may elicit a similar effect on the cornea or the muzzle through hemolysin, or other biological determinants, that is not fully apparent to human eyes. Additional research could address this possibility, and facilitate calibration of the AI system to specific IBK pathogens and/or virulence factors.

How far can we push AI for disease detection in cattle? Many cattle diseases, like IBK, are polymicrobial, which gives the possibility of different muzzle phenotypes for the same disease. One of the most important diseases of cattle worldwide is BRD, which is both multifactorial and polymicrobial. The AI system is currently being trained to predict BRD by muzzle profiles, and early results indicate that it can distinguish emerging cases, although more training of the system with larger numbers of animals will be required to match the sensitivity and specificity of detection for IBK. Ultimately, AI may be incorporated as a vital tool for precision management of cattle, ensuring maximal health and well-being as part of the production process.

## Data Availability

Cattle muzzle pictures in the MyAnIML database that were used to train MyAnIML’s proprietary AI system are not publicly available. Data from the IBK study are available from corresponding author Shekhar Gupta upon reasonable request.
